# Supplementary material for: NMT1 and NMT2 are lysine myristoyltransferases regulating the ARF6 GTPase cycle
Source: Nat Commun. 2020 Feb 26;11:1067. doi: 10.1038/s41467-020-14893-x (PMC7044312; doi:10.1038/s41467-020-14893-x)
Supplement: Supplementary file 1 — Supplementary Information [file 41467_2020_14893_MOESM1_ESM.pdf]

Supplementary Information

NMT1 and NMT2 are Lysine Myristoyltransferases Regulating the ARF6 GTPase Cycle

Tatsiana Kosciuk[1], Ian R. Price[1], Xiaoyu Zhang[1], Chengliang Zhu[1], Kayla N. Johnson[1], Shuai Zhang[1,2], Steve L. Halaby[3], Garrison P. Komaniecki[1], Min Yang[1], Caroline J. DeHart[4], Paul M. Thomas[4], Neil L. Kelleher[4], J. Christopher Fromme[3], Hening Lin[1,2]

[1]Department of Chemistry and Chemical Biology, Cornell University, Ithaca, NY 14853, USA

[2]Howard Hughes Medical Institute; Department of Chemistry and Chemical Biology, Cornell University, Ithaca, NY 14853, USA

[3]Department of Molecular Biology and Genetics; Weill Institute for Cell and Molecular Biology, Cornell University, Ithaca, NY 14853, USA

[4]National Resource for Translational and Developmental Proteomics, Departments of Chemistry, Molecular Biosciences and the Feinberg School of Medicine, Northwestern University, Evanston, IL 60208, USA

Supplementary Table 1: Data collection and refinement statistics. Statistics for the highest-resolution shell are shown in parentheses.

	NMT2 with myristoyl-KVLSKIF and CoA (PDB: 6PAU)	NMT1 with myristoyl-AcKVLSKIF and CoA (PDB: 6PAV)
**Wavelength (Å)**	0.97918	0.97918
**Resolution range (Å)**	57.59 - 1.93 (2.00 - 1.93)	51.34 - 2.52 (2.61 - 2.52)
**Space group**	P 1 21 1	C 1 2 1
**Unit cell** a/b/c (Å):<br>α/β/γ:	63.16/ 46.35/ 74.40<br>90°/ 114.25°/ 90°	92.36/ 58.22/ 154.04<br>90°/ 90.66°/ 90°
**Total reflections**	154166 (15911)	104948 (9898)
**Unique reflections**	29746 (2966)	27918 (2722)
**Multiplicity**	5.2 (5.3)	3.8 (3.6)
**Completeness (%)**	99.49 (99.56)	98.45 (97.21)
**Mean I/sigma(I)**	11.35 (1.54)	4.98 (0.94)
**Wilson B-factor**	29.29	51.51
**R-merge**	0.1007 (1.066)	0.3053 (1.196)
**R-meas**	0.1123 (1.184)	0.3558 (1.404)
**R-pim**	0.04867 (0.505)	0.1804 (0.7275)
**CC1/2**	0.998 (0.646)	0.919 (0.34)
**CC***	0.999 (0.886)	0.979 (0.713)
**Reflections used in refinement**	29687 (2965)	27560 (2719)
**Reflections used for R-free**	1529 (161)	1468 (158)
**R-work**	0.1973 (0.3721)	0.2644 (0.4147)
**R-free**	0.2174 (0.4192)	0.2819 (0.4281)
**CC(work)**	0.963 (0.841)	0.913 (0.615)
**CC(free)**	0.956 (0.754)	0.900 (0.517)
**Number non-hydrogen atoms**	3320	6521
**macromolecules**	3043	6287
**ligands**	101	150
**solvent**	176	84
**Protein residues**	369	771
**RMS(bonds)**	0.008	0.012
**RMS(angles)**	1.20	1.66
**Ramachandran favored (%)**	96.70	96.57
**Ramachandran allowed (%)**	3.30	3.43
**Ramachandran outliers (%)**	0.00	0.00
**Rotamer outliers (%)**	0.29	1.58
**Clashscore**	5.52	13.53
**Average B-factor**	37.87	58.61
**macromolecules**	37.09	58.29
**ligands**	54.37	80.85
**solvent**	41.79	42.99
**DPI (Å)**	0.169	0.343

Supplementary Table 2: Primers used for cloning and squencing.

**ARF6 and ARF1 mutagenesis**	
Arf6_G2A+A_F	GATATCGGTACCATG**GCAGCG**AAGGTGCTATCC
Arf6_G2A+A_R	GGATAGCACCTTCGCTGCCATGGTACCGATATC
Arf6_G2A+G_F	ATCGGTACCATGGCG**GGG**AAGGTGCTATCCAAG
Arf6_G2A+G_R	CTTGGATAGCACCTTCCCTGCCATGGTACCGAT
Arf6_G2A+2A_F	GATATCGGTACCATG**GCTGCAGCG**AAGGTGCTATCC
Arf6_G2A+2A_R	GGATAGCACCTTCGCTGCAGCCATGGTACCGATATC
Arf6_Q67L_F	tgggatgtgggcggc**ctg**gacaagatccggccg
Arf6_Q67L_R	gccgcccacatcccacacgttgaacttgacgtt
Arf6 T27N_F	gacgcagccggcaag**aac**acgatcctgtacaag
Arf6 T27N_R	cttgccggctgcgtccaggcccagcatgaggat
Arf6_G2A_F	GATATCGGTACCATG**GCG**AAGGTGCTATCCAAG
Arf6_G2A_R	CATGGTACCGATATCAGATCTATCGATGAATTC
Arf6_G2A_K3R_F	GATATCGGTACCATG**GCGAGG**GTGCTATCCAAGATC
Arf6_G2A_K3R_R	CATGGTACCGATATCAGATCTATCGATGAATTC
Arf1_G2A_F	GATATCGGTACCATG**GCG**AATATCTTTGCAAAC
Arf1_G2A_R	CATGGTACCGATATCAGATCTATCGATGAATTC
Arf1 g2a/n3k_F	ATCGGTACCATG**GCG**AAAATCTTTGCAAACCTC
Arf1 g2a/n3k_R	CGCCATGGTACCGATATCAGATCTATCGATGAA
**NMT cloning into pCMV4a vector**	
NMT1-5'-ecor1-ha	caattatat**gaattc**atgTACCCATACGATGTTCCAGATTACGCTggagcggacgagagtgagacagcagtg
NMT1-3'-xho1-stop	caattatat**ctcgag**ttattgtagcaccagtccaaccttctctg
NMT2-5'-ecor1-ha	caattatat**gaattc**atgTACCCATACGATGTTCCAGATTACGCTggagcggaggacagcgagtctg
NMT2-3'-xho1-stop	caattatat**ctcgag**ctattgtagtactagtccaaccttttcagaatctgtacctgg
**Full length NMT cloning into pETHisTEV (Gibson assembly)**	
NMT1_F	GCAGCCATCATCATCATCACATGGCGGACGAGAGTGAG
NMT1_R	AAATACAGGTTTTCGCTAGCTTATTGTAGCACCAGTCCAACC
NMT2_F	GCAGCCATCATCATCATCACATGGCGGAGGACAGCGAG
NMT2_R	AAATACAGGTTTTCGCTAGCCTATTGTAGTACTAGTCCAACCTTTTCAGAATCTG
**Mutagenesis to add His residue to the 5xHis tag**	
his-f	TATACCATGGGCAGC**CAC**CATCATCATCATCAC
his-r	GCTGCCCATGGTATATCTCCTTCTTAAAGTTAA
**NMT catalytic domains cloning into pETHisTEV (Gibson assembly)**	
NMT1 Forward	(CCATGG) GCAGCAGCCATCATCATCATCATCACAGCAGCGGC CTTGAAGTCCTCTT TCAGGGACCC CATATG
NMT1 Reverse	gtcgacggagctcgaattcggatccTTATTGTAGCACCAGTCCAACC
NMT2 Forward	(CCATGG) GCAGCAGCCATCATCATCATCATCACAGCAGCGGC CTTGAAGTCCT CTTTCAGGGACCC CATATG
NMT2 Reverse	gtcgacggagctcgaattcggatccCTATTGTAGTACTAGTCCAACCTTTTC
**qRT-PCR primers**	
NMT1_F	GGTCAGGGACCTGCCAAAAC
NMT1_R	CATGGGTGTTCACCACTTCG
NMT2_F	TCCCAGCAAACATTCGGATTT
NMT2_R	ACCCGTTTCGATCTCAACTTCT

HDAC11_F	CACGCTCGCCATCAAGTTTC
HDAC11_R	GAAGTCTCGCTCATGCCCATT
GAPDH_F	ACAACTTTGGTATCGTGGAAGG
GAPDH_R	GCCATCACGCCACAGTTTC

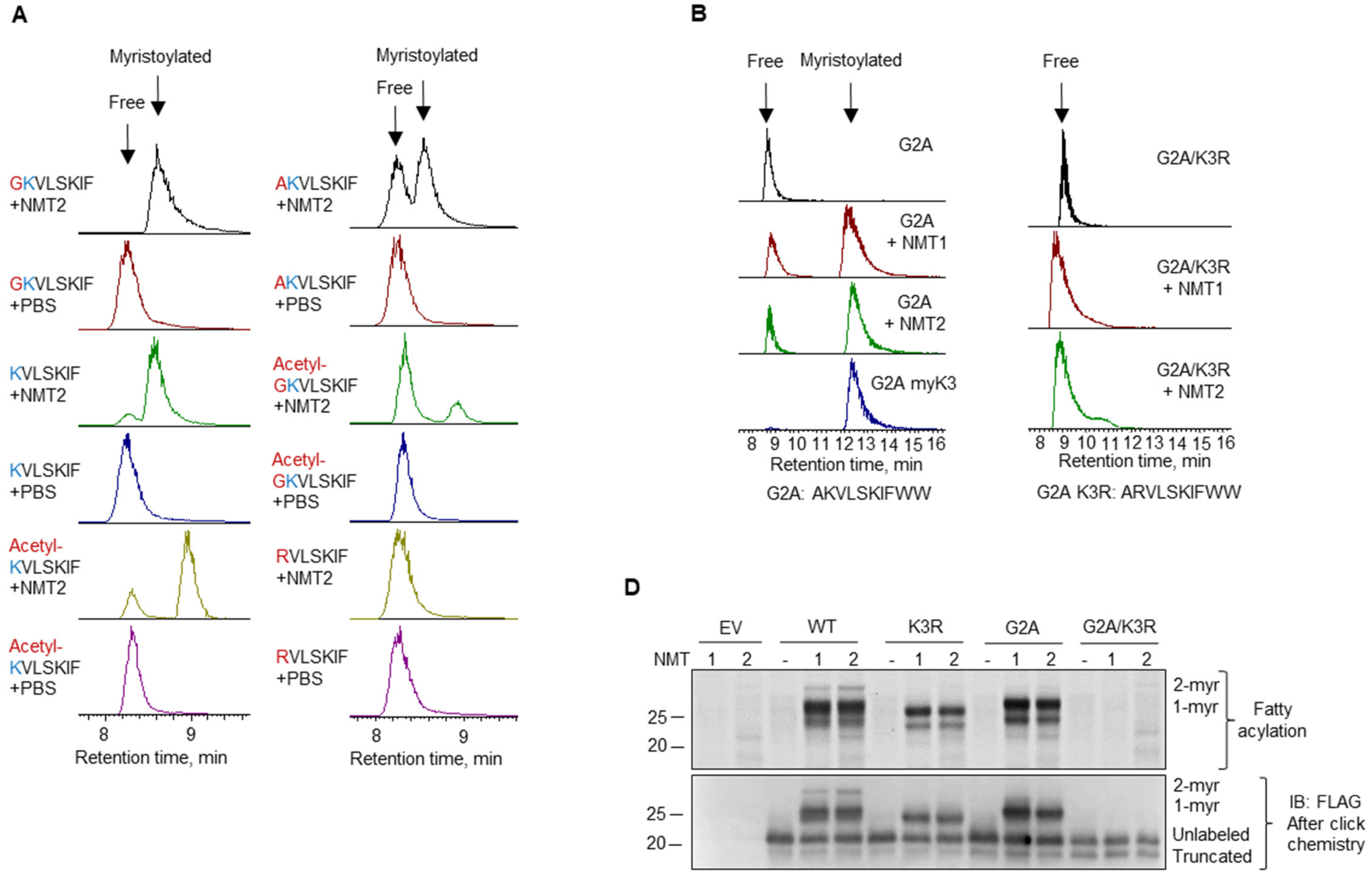

C

NMT1

Peptide	*K_m, (μM)*	*k_{cat}, (s^{-1})*	*k_{cat}/K_m, ($s^{-1}M^{-1}$)*
ARF6 K3R	7.19 ± 0.48	(30.65 ± 0.81)X10^{-4}	426 ± 167
ARF6 G2A	5.31 ± 2.84	(7.62 ± 0.17)X10^{-4}	144 ± 6

NMT2

Peptide	*K_m, (μM)*	*k_{cat}, (s^{-1})*	*k_{cat}/K_m, ($s^{-1}M^{-1}$)*
ARF6 K3R	14.28 ± 2.83	(36.46 ± 1.59)X10^{-4}	252 ± 56
ARF6 G2A	6.73 ± 0.14	(8.95 ± 0.07)X10^{-4}	133 ± 16

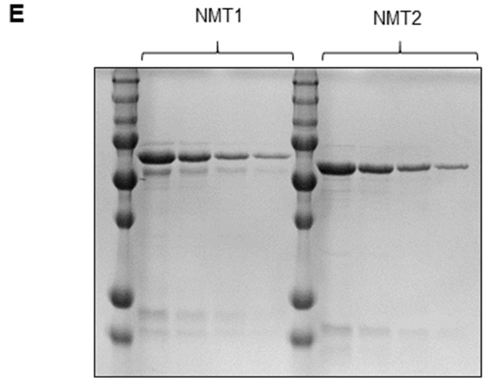

Supplementary Figure 1: NMT myristoylates K3 of ARF6. (A) Mass traces of NMT2 reactions on ARF6 synthetic peptides are shown. (B) NMT cannot myristoylate G2A/K3R peptide. (C) NMT kinetics on indicated peptides. (D) In vitro NMT reaction with ALK12-CoA on ARF6 mutants suggests multiple acylation products. (E) Protein gel showing the purity of full-length recombinant NMT1 and NMT2.

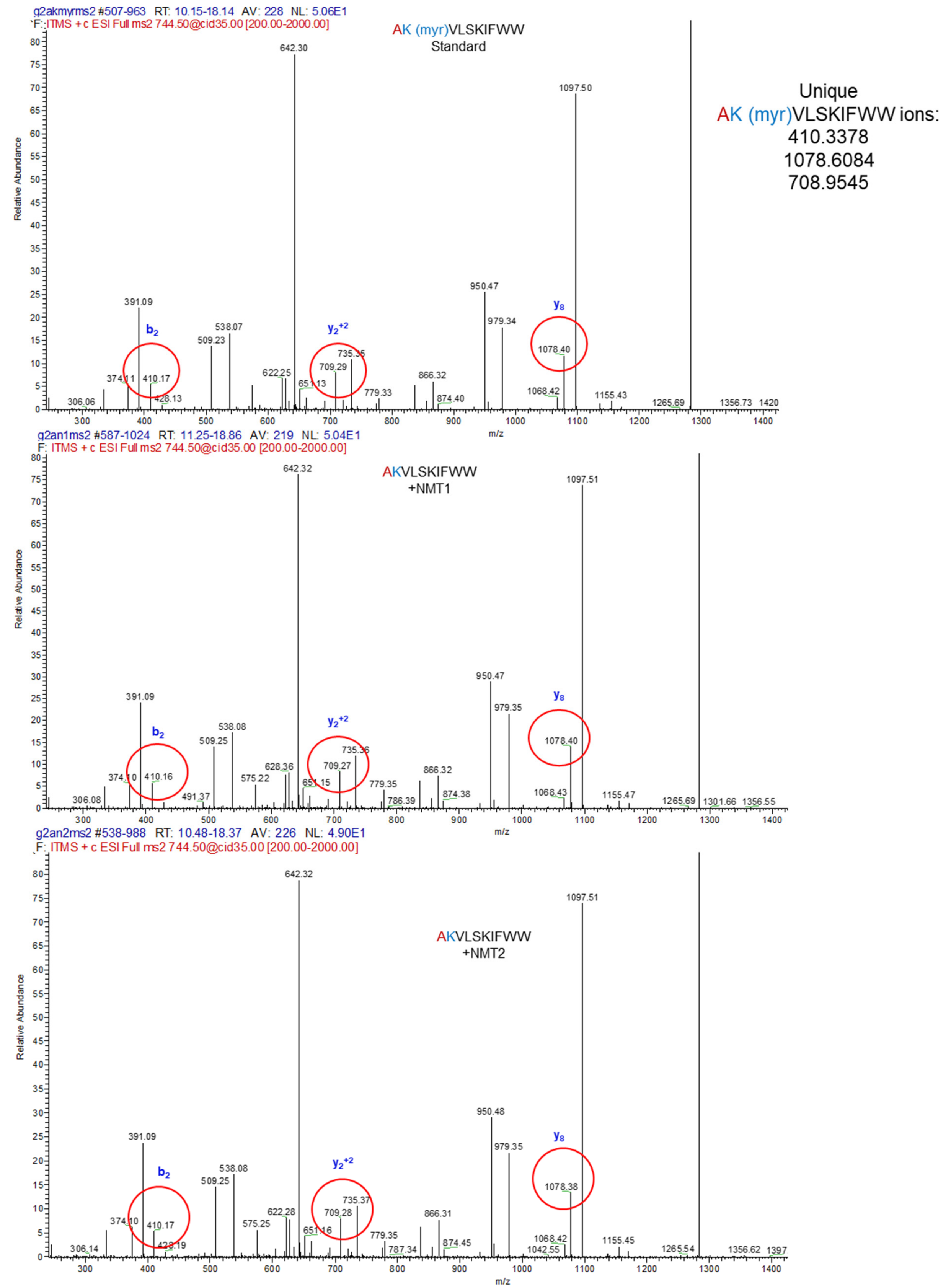

Supplementary Figure 2: NMT1 and NMT2 modify ARF6 G2A peptide on K3. Tandem MS spectra of standard myristoyl peptide and products from NMT reactions are shown. Circled fragment ions are unique to the acylated product while others are common to both substrate and product.

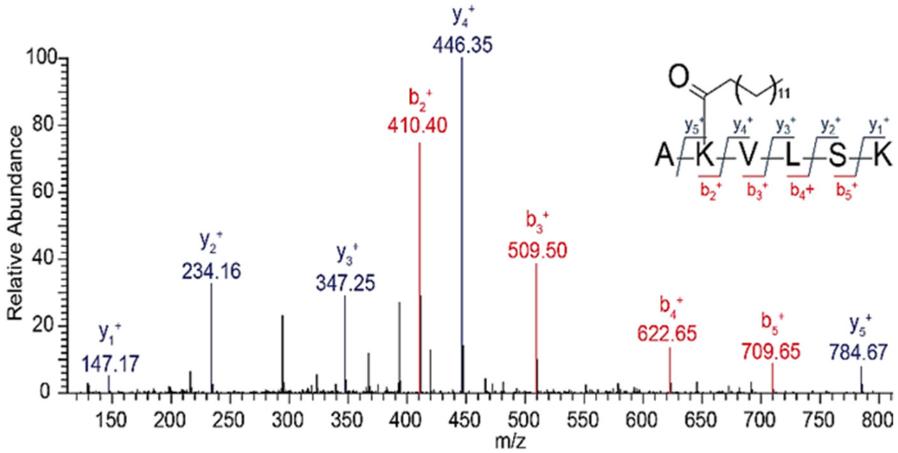

Supplementary Figure 3: K3 myristoylation on ARF6 G2A identified by MS/MS. ARF6 G2A mutant was isolated from SIRT2 KD HEK 293T cells.

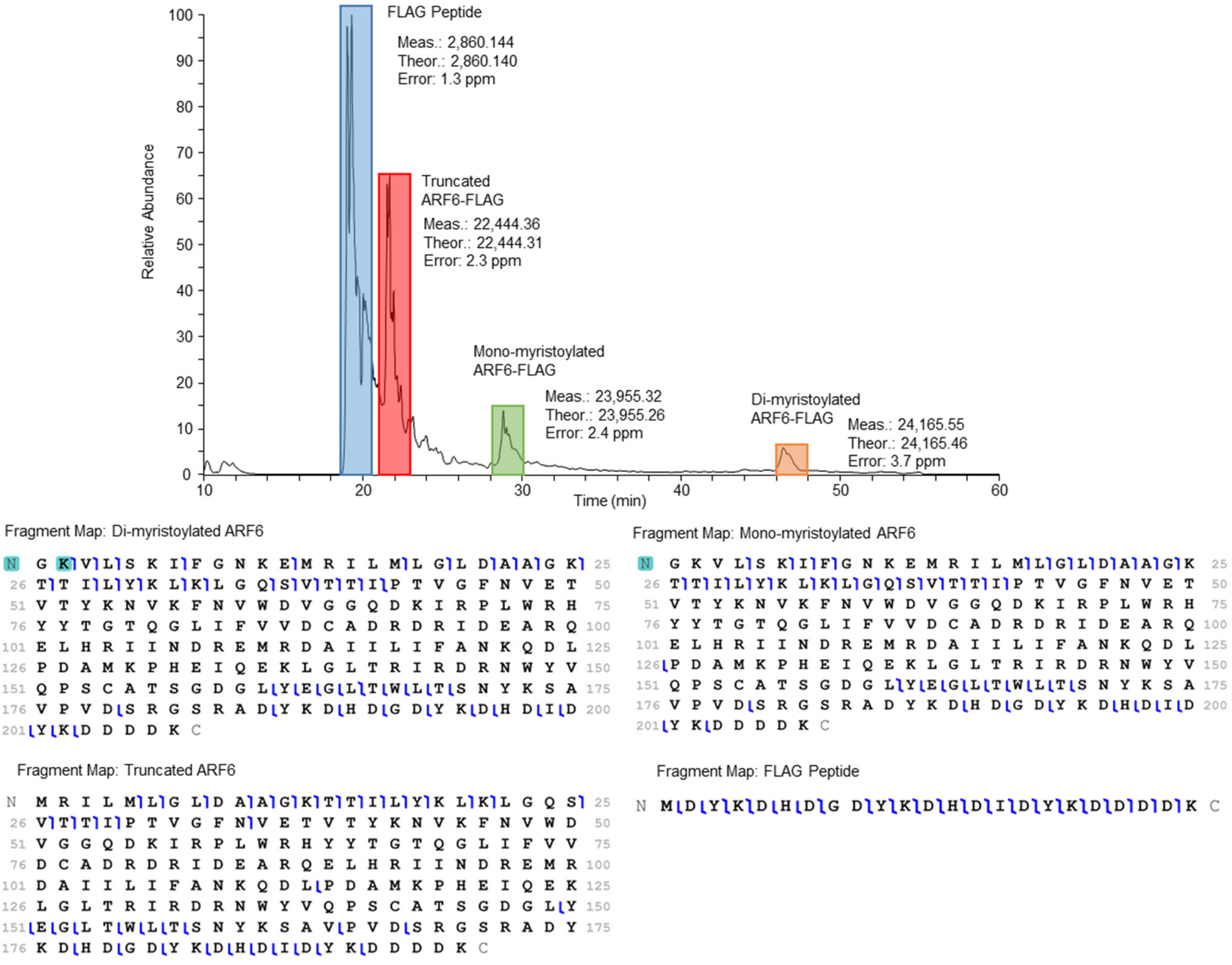

Supplementary Figure 4: Di-myristoylated ARF6 is identified by top-down mass spectrometry. Annotated chromatogram showing identified protein species and corresponding fragment maps. Highlighted N and K indicate the modification sites at the N-terminus and lysine residue. Flag peptide was used to elute the protein during sample preparation. The truncated species is caused by the alternative start site and explains the lower band observed by western blot.

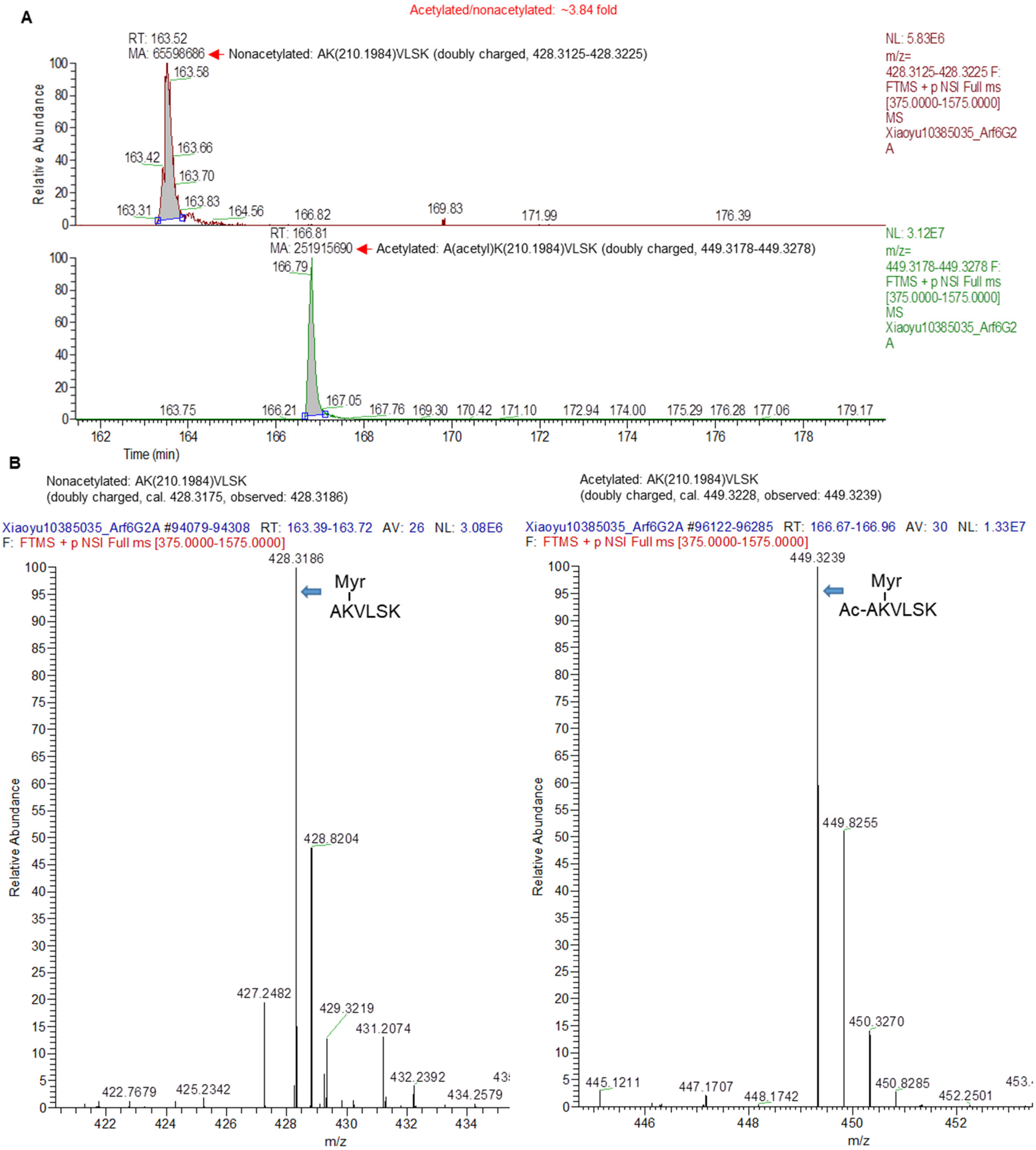

Supplementary Figure 5: Comparison of N-terminal acetylated vs. nonacetylated ARF6 G2A K3 myristoylated sequence. A) TIC and peak quantification showing that the peptide that is both acetylated and myristoylated is more abundant than the myristoylated only peptide. B) Mass spectra for the N-terminal peptides of ARF6 G2A.

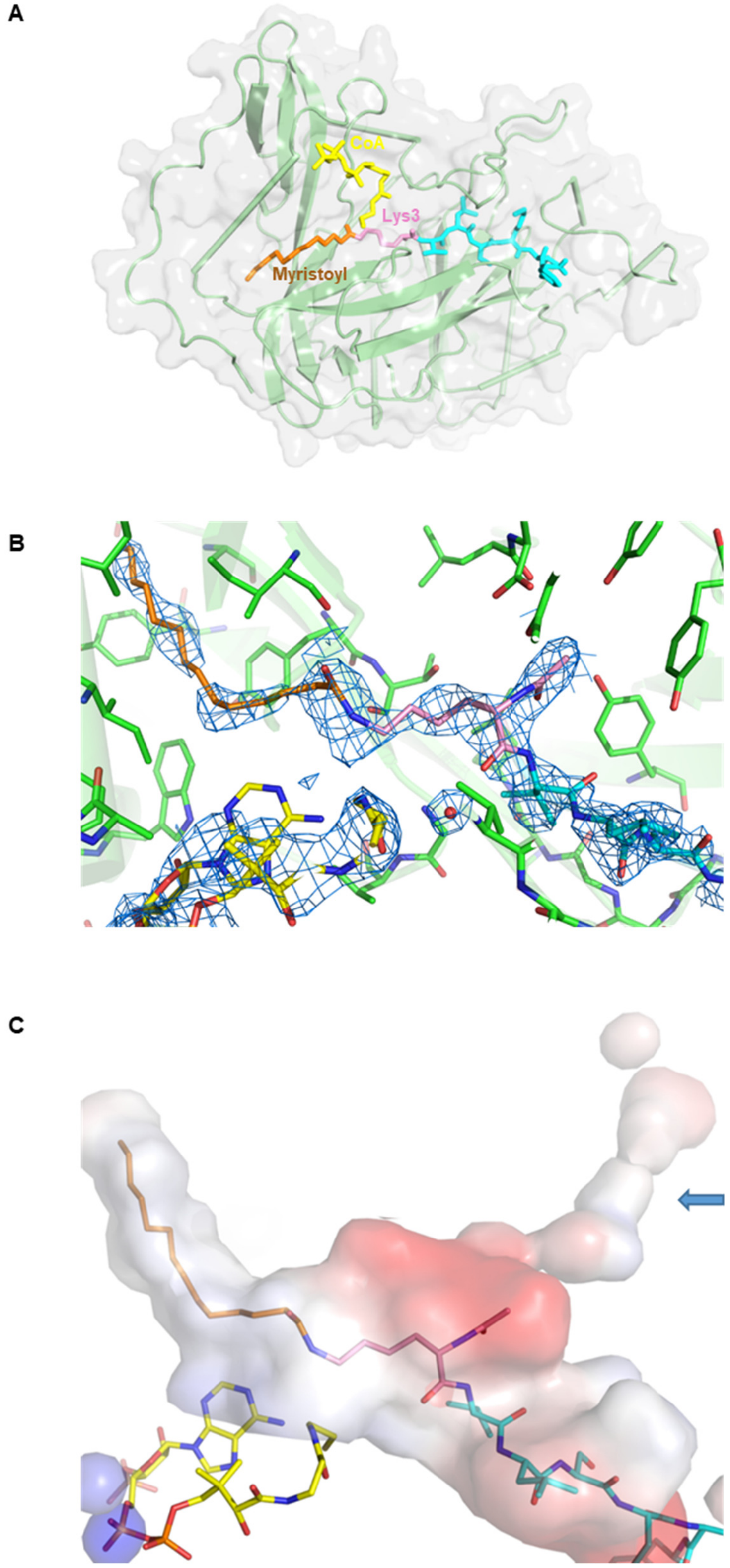

Supplementary Figure 6: (A) Overall structure of NMT2 catalytic domain with myristoyl-KVLSKIF peptide and CoA. (B) NMT1-AcKVLSKIF, $2F_O$-F_C simulated annealing omit map, 1.0 σ. (C) Interior pockets (shown as surface) around the NMT1 active site. The surface is colored by the electrostatic properties of the surrounding residues: blue (positive), red (negative), and gray (hydrophobic). Blue arrow points to the hydrophobic pocket.

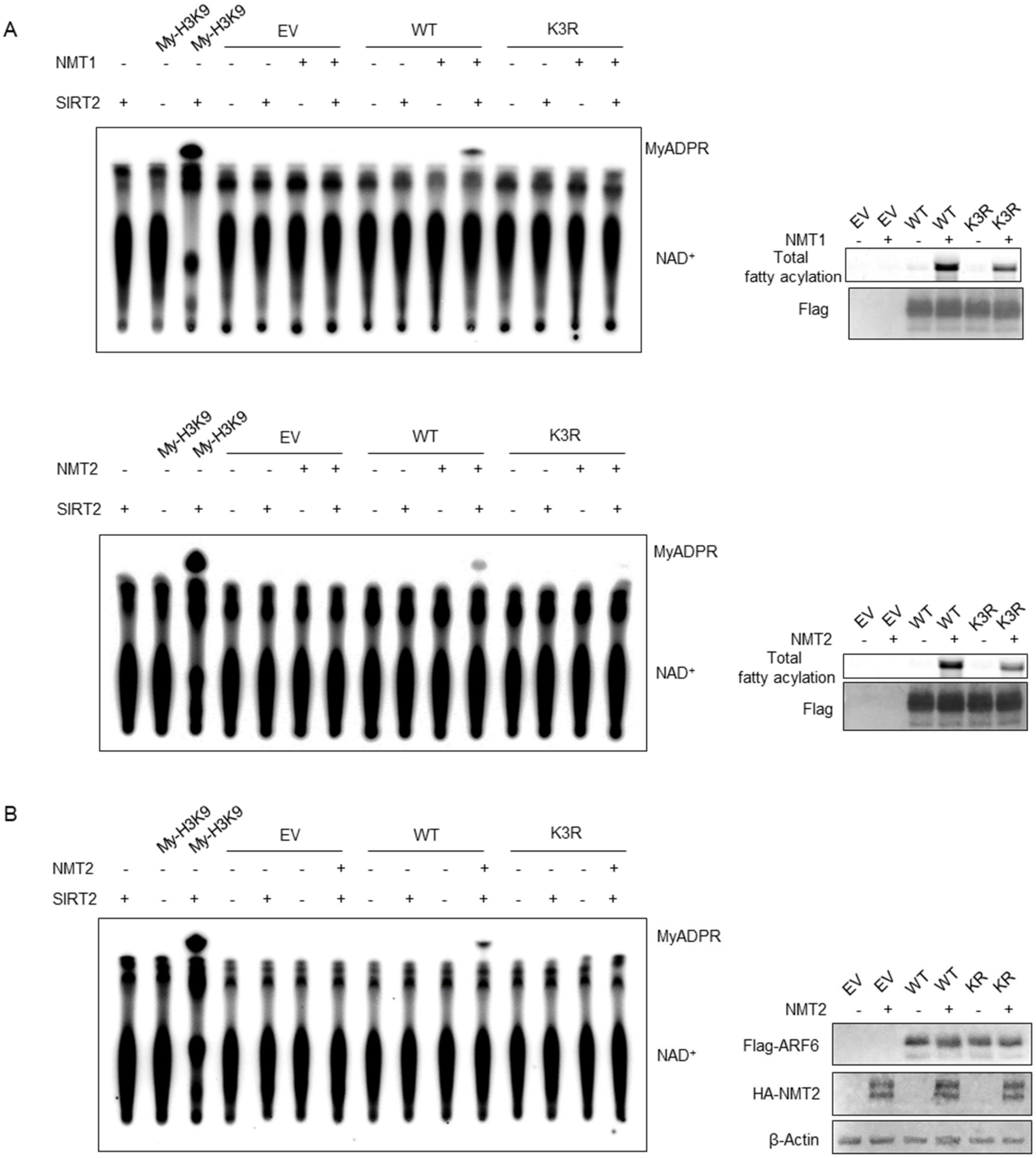

Supplementary Figure 7: ^{32}P-NAD$^+$ assays showing that NMT1 and NMT2 modify ARF6 WT on K3. (A) ARF6 WT or K3R mutant were modified with NMT1 or NMT2 in vitro, and then subjected to ^{32}P-NAD$^+$ assay to detect lysine myristoylation. Total myristoylation levels (detected by Alk12-CoA labeling and in-gel fluorescence) and western blots showing protein levels are shown on the right. (B) NMT2 myristoylates ARF6 WT on K3 in cells. ARF6 WT and K3R were purified from HEK293T cells with or without NMT2 overexpression, and then subjected to ^{32}P-NAD$^+$ assay to detect lysine myristoylation. Western blots showing protein levels are shown on the right. One replicate for A and B.

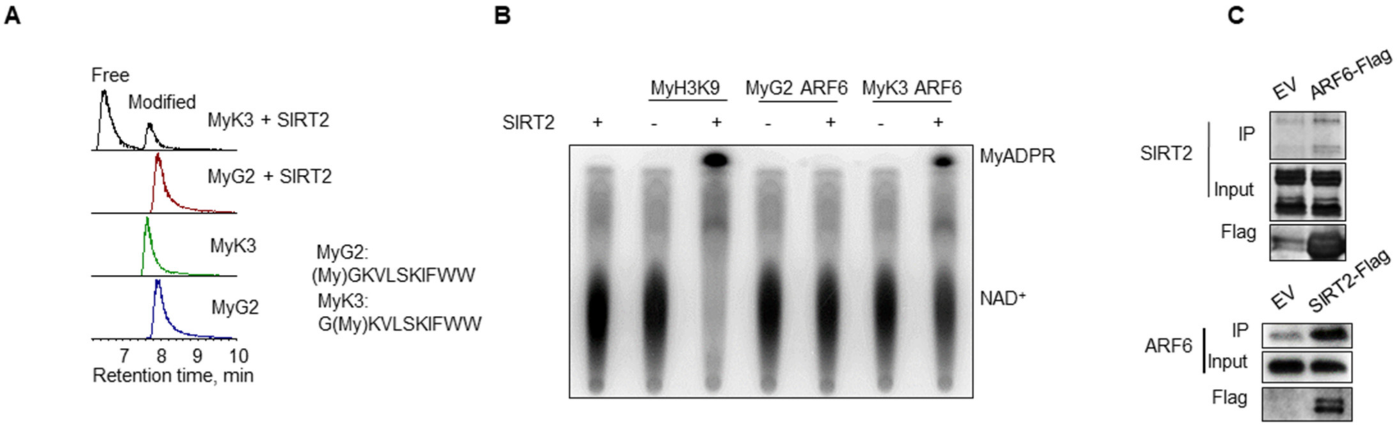

Supplementary Figure 8: SIRT2 demyristoylates ARF6 K3. (A) LC-MS traces of SIRT2 reactions on myristoylated ARF6 N-terminal peptides showing that SIRT2 demyristoylates K3 but not G2. (B) P^{32}-NAD^{+} assay on ARF6 N-terminal peptides myristoylated on G2 or K3 showing that MyADPR is formed with the addition of SIRT2 to K3 but not G2 myristoylated peptides. (C) SIRT2 and ARF6 associate with each other as detected by co-IP.

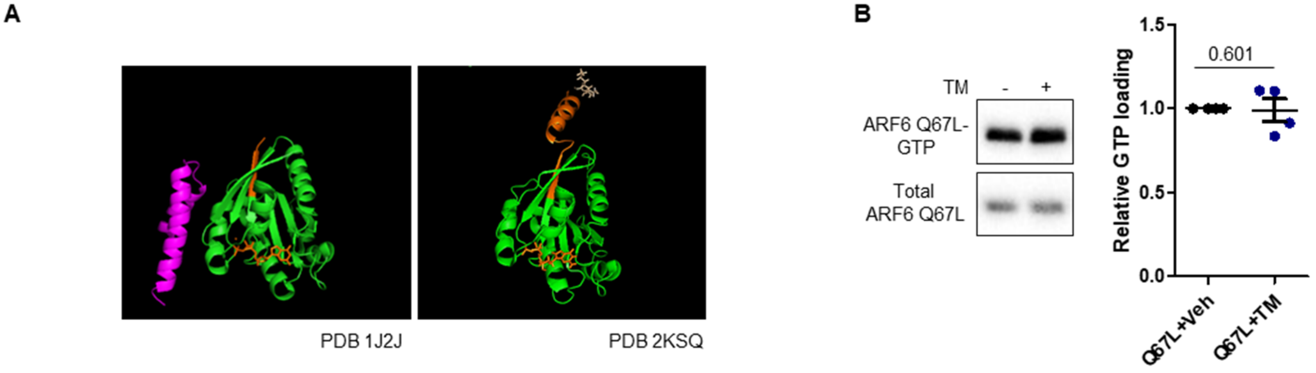

Supplementary Figure 9: ARF6 lysine 3 myristoylation is unlikely to affect GGA3 binding. (A) Crystal structures of ARF1-GTP without N-terminal helix in complex with binding domain of GGA1 (PDB 1J2J) and ARF1-GTP with myristoylated N-terminal helix (PDB 2KSQ). ARF1 – green, GGA1 – cyan, GTP and N-terminal helix with preceding residues – orange. The myristoylated N-terminal helix is far from the binding domain of GGA1. (B) TM does not affect GGA3 binding to ARF6 Q67L, suggesting that lysine 3 myristoylation does not affect ARF6 binding to GGA3. Error bars represent SEM.

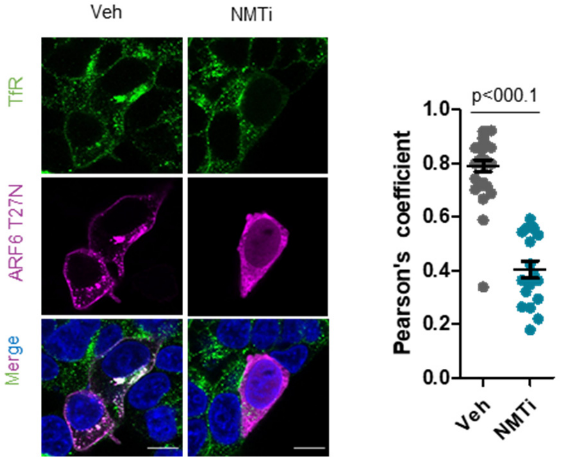

Supplementary Figure 10: NMTi inhibits ARF6 T27N colocalization with TfR. Each point in the right quantification plot represents one cell (Veh=27, NMTi=17), quantification shows Pearson's correlation coefficient with SEM. Scale bars: 10 µM. (n=1).

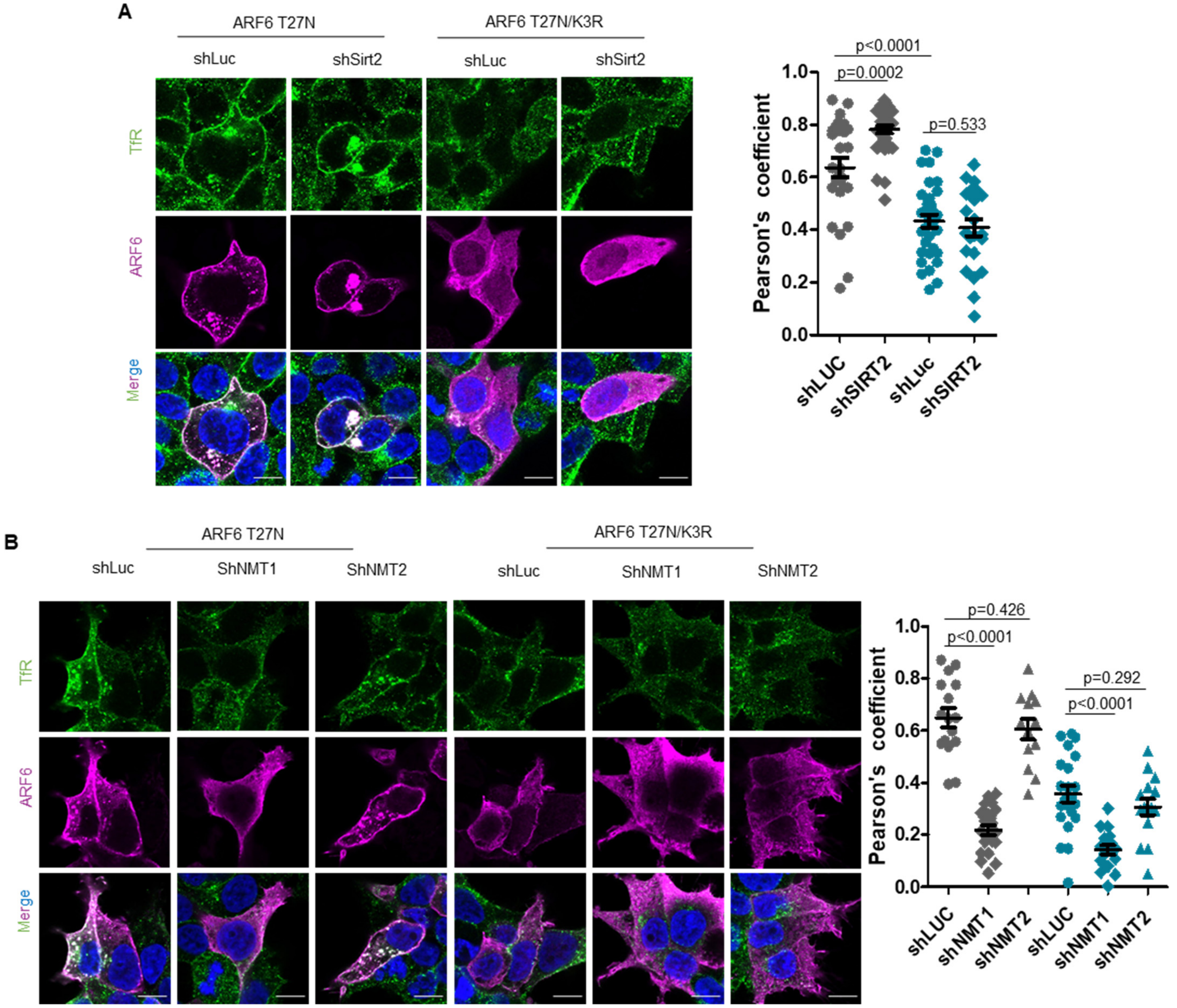

Supplementary Figure 11: ARF6 lysine myristoylation cycle regulated membrane localization of inactive ARF6. (A) SIRT2 KD promotes ARF6 T27N (but not T27N/K3R) association with membranes as indicated by colocalization with TfR. (B) NMT1 KD inhibits ARF6 T27N colocalization with TfR. Both A and B were performed in HEK293T cells transiently overexpressing ARF6 T27N. n=3 and n=2 for SIRT2 KD and NMT KD respectively. Scale bars: 10 μM. Each point is one cell, (for (A) shLuc/T27N=27, shSIRT2/T27N=34, shLuc/T27N/K3R=33, shSIRT2/T27N/K3R=23; for (B) shLuc/T27N=16, shNMT1/T27N=22, shNMT2/T27N=13, shLuc/T27N/K3R=22, shNMT1/T27N/K3R=17, shNMT2/T27N/K3R=15) Quantifications represent Pearson's correlation coefficients with SEM. Unpaired two-tailed t-test.

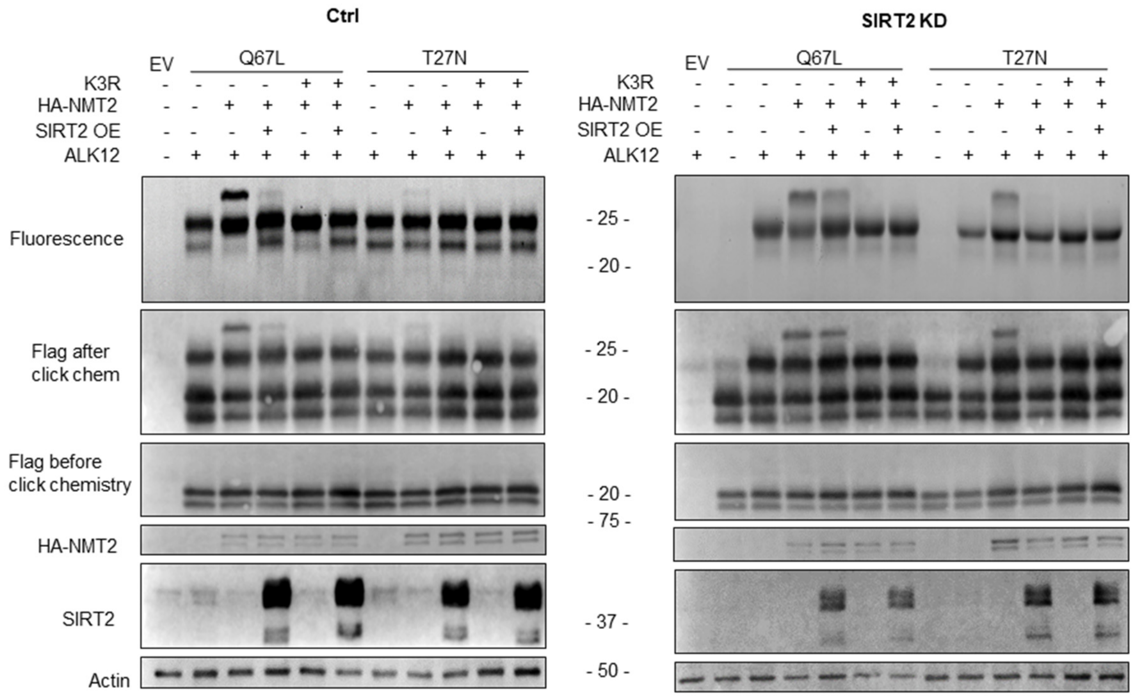

Supplementary Figure 12: NMT and SIRT2 prefer different catalytic states of ARF6. In-cell Alk12 labeling of ARF6 Q67L and T27N mutants with NMT2 OE and/or SIRT2 OE/KD showing more lysine acylation of ARF6 Q67L.

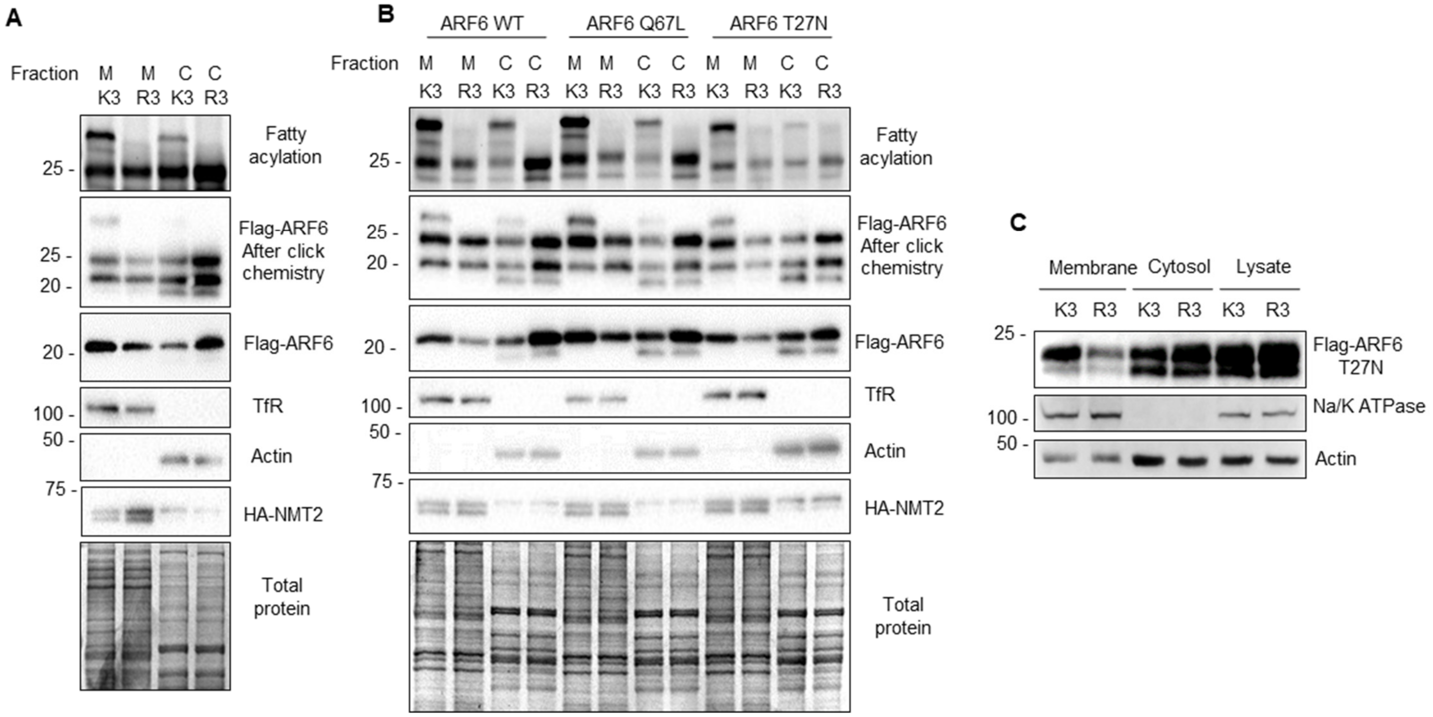

Supplementary Figure 13: K3 myristoylation promotes ARF6 membrane localization. (A) ARF6 K3 myristoylation promotes membrane association of ARF6 WT in HEK 293T cells. (B) ARF6 K3 myristoylation promotes membrane association of active and inactive mutants of ARF6 in HEK 293T cells. SIRT2 KD cells were used for this experiment. Cell overexpressing ARF6 WT and mutants were subjected to differential centrifugation. (C) ARF6 T27N fractionation in HEK293T cells without NMT OE showing that abrogating K3 myristoylation with K3R mutation inhibits membrane localization of inactive ARF6.

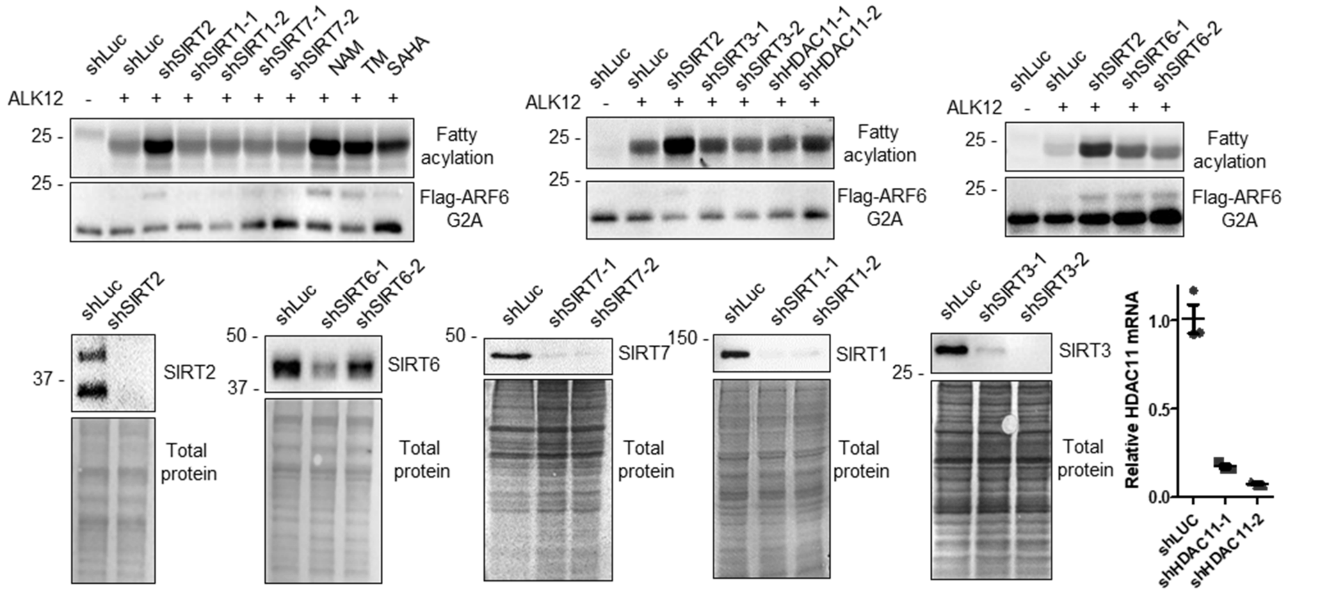

Supplementary Figure 14: SIRT2 but not other lysine defatty acylases regulate ARF6 lysine fatty acylation. ARF6 G2A was overexpressed in HEK293T cells with stable KD of indicated proteins. HDAC11 KD efficiency was analyzed by qRT-PCR due to lack of a band of expected molecular weight by Western Blot. Error bars represent SEM from three technical replicates analyzed by unpaired two-tailed t-test.

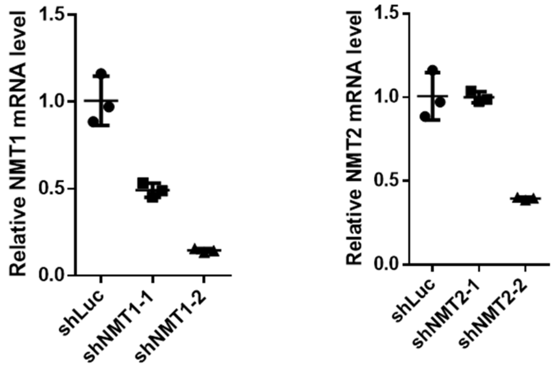

Supplementary Figure 15: NMT KD efficiency determined by qRT-PCR. This experiment was done several months later than the Western blot results shown in Fig. 2C and over time the knockdown efficiency of shNMT2-1 decreased. Error bars represent SEM from three technical replicates analyzed by unpaired two-tailed t-test.

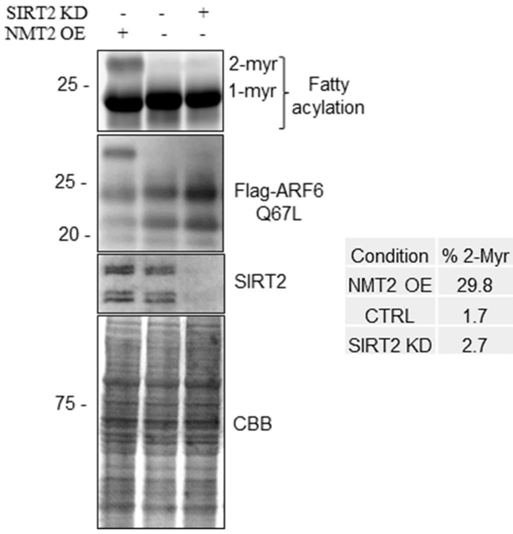

Condition	% 2-Myr
NMT2 OE	29.8
CTRL	1.7
SIRT2 KD	2.7

Supplementary Figure 16: Endogenous NMT can dimyristoylate ARF6-GTP. ALK12 labeling performed in HEK293T cells transiently overexpressing ARF6 Q67L. The percentage of dimyristoylation was quantified using ImageJ.

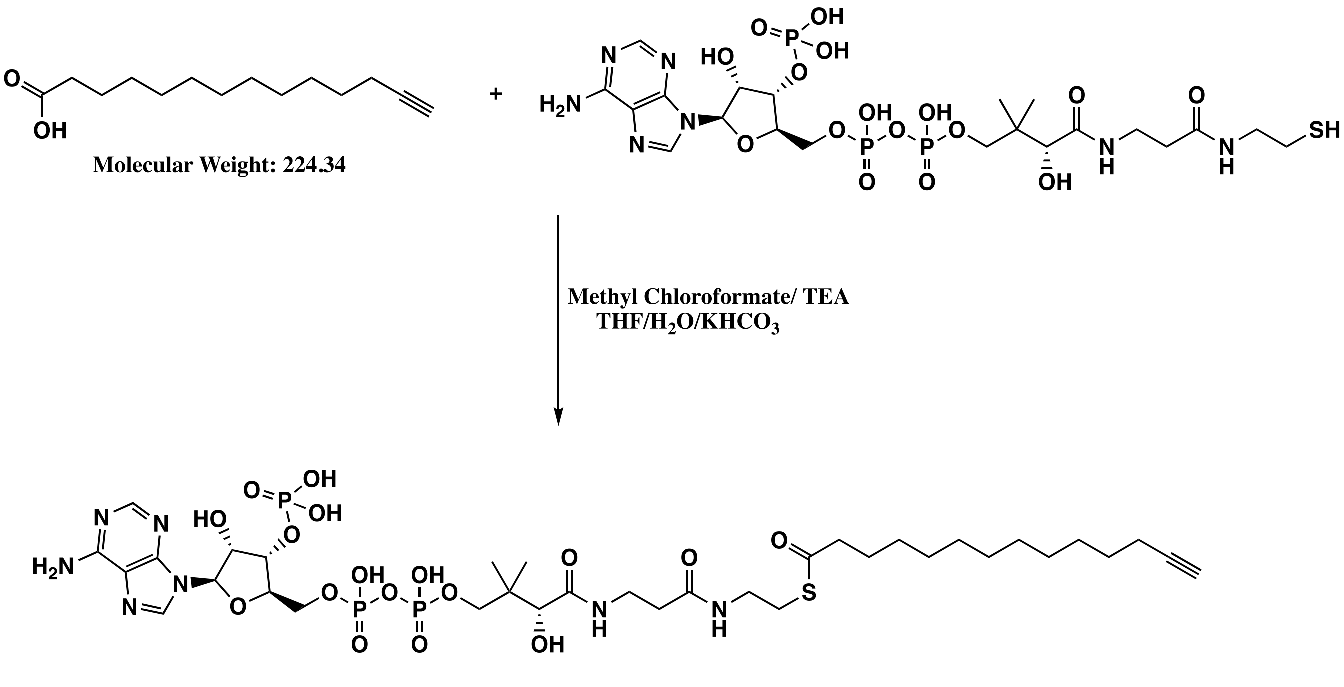

Supplementary Figure 17: ALK12-CoA synthesis scheme.
