# Supplementary material for: NMT1 and NMT2 are lysine myristoyltransferases regulating the ARF6 GTPase cycle
Source: Nat Commun. 2020 Feb 26;11:1067. doi: 10.1038/s41467-020-14893-x (PMC7044312; doi:10.1038/s41467-020-14893-x)
Supplement: Supplementary file 3 — Reporting Summary [file 41467_2020_14893_MOESM3_ESM.pdf]

nature research

Corresponding author(s): Hening Lin

Last updated by author(s): Jan 26, 2020

Reporting Summary

Nature Research wishes to improve the reproducibility of the work that we publish. This form provides structure for consistency and transparency in reporting. For further information on Nature Research policies, see Authors & Referees and the Editorial Policy Checklist .

Statistics

For all statistical analyses, confirm that the following items are present in the figure legend, table legend, main text, or Methods section.

n/a	Confirmed	
☐	☒	The exact sample size (*n*) for each experimental group/condition, given as a discrete number and unit of measurement
☐	☒	A statement on whether measurements were taken from distinct samples or whether the same sample was measured repeatedly
☐	☒	The statistical test(s) used AND whether they are one- or two-sided<br>*Only common tests should be described solely by name; describe more complex techniques in the Methods section.*
☒	☐	A description of all covariates tested
☒	☐	A description of any assumptions or corrections, such as tests of normality and adjustment for multiple comparisons
☐	☒	A full description of the statistical parameters including central tendency (e.g. means) or other basic estimates (e.g. regression coefficient) AND variation (e.g. standard deviation) or associated estimates of uncertainty (e.g. confidence intervals)
☐	☒	For null hypothesis testing, the test statistic (e.g. *F*, *t*, *r*) with confidence intervals, effect sizes, degrees of freedom and *P* value noted<br>*Give P values as exact values whenever suitable.*
☒	☐	For Bayesian analysis, information on the choice of priors and Markov chain Monte Carlo settings
☒	☐	For hierarchical and complex designs, identification of the appropriate level for tests and full reporting of outcomes
☐	☒	Estimates of effect sizes (e.g. Cohen's *d*, Pearson's *r*), indicating how they were calculated

Our web collection on statistics for biologists contains articles on many of the points above.

Software and code

Policy information about availability of computer code

Data collection	For crystalography work datasets were collected at the NE-CAT beamline 24-ID-E at the Advanced Photon Source (Supplementary Table 1). Images were indexed, integrated, and merged using XDS and Aimless in the RAPD pipeline at NE-CAT and further cut according to CC1/2 and I/sigmal statistics in Phenix6. Structures were solved using PHASER molecular replacement 7. Models were constructed using iterative building in COOT8 and refinement in Phenix9.
Data analysis	Graph Pad Prism 5 or 6 was used for statistical analysis and Fiji and JACop plugin was used for colocalization analysis.

For manuscripts utilizing custom algorithms or software that are central to the research but not yet described in published literature, software must be made available to editors/reviewers. We strongly encourage code deposition in a community repository (e.g. GitHub). See the Nature Research guidelines for submitting code & software for further information.

Data

Policy information about availability of data

All manuscripts must include a data availability statement. This statement should provide the following information, where applicable:

- Accession codes, unique identifiers, or web links for publicly available datasets
- A list of figures that have associated raw data
- A description of any restrictions on data availability

All figures have associated raw data that can be provided by the corresponding author upon a reasonable request or they are provided in the source data file.

Field-specific reporting

Please select the one below that is the best fit for your research. If you are not sure, read the appropriate sections before making your selection.

Life sciences study design

All studies must disclose on these points even when the disclosure is negative.

Sample size	Cell based experiment were done in at least two distinct replicates, which is sufficient to study the effects of fatty acylation. The number of images was chosen based on the transfection efficiency.
Data exclusions	Two values were excluded from Supplementary Figure 1C calculations for more appropriate Michaelis-Menten fit. This exclusion did not strongly impact the resulting values. The exclusion is noted in the source data file.
Replication	Ideas were tested with similar approaches that led to similar conclusions. Most experiments were done in at least two biological replicates. Key experiments were replicated by several authors.
Randomization	Samples were processed in a random order to avoid systematic error.
Blinding	N/A

Reporting for specific materials, systems and methods

We require information from authors about some types of materials, experimental systems and methods used in many studies. Here, indicate whether each material, system or method listed is relevant to your study. If you are not sure if a list item applies to your research, read the appropriate section before selecting a response.

Materials & experimental systems

n/a	Involved in the study	
☐	☒	Antibodies
☐	☒	Eukaryotic cell lines
☒	☐	Palaeontology
☒	☐	Animals and other organisms
☒	☐	Human research participants
☒	☐	Clinical data

Methods

n/a	Involved in the study	
☒	☐	ChIP-seq
☒	☐	Flow cytometry
☒	☐	MRI-based neuroimaging

Antibodies

Antibodies used	Anti-FLAG affinity gel (#A2220, RRID: AB_10063035) and FLAG-HRP (#A8592, RRID: AB_439702) were purchased from Sigma. HA-HRP (sc-7392), Na/K-ATPase (C464.6, sc-21712), β-Actin-HRP (C4, sc-47778), NMT1 (E-9, sc-393702), NMT2 (30, sc-136005), TfR (CD-71) (3B8 2A1, sc-32272), ARF6 (3A-1, sc-7971), antibodies were purchased from Santa Cruz Biotechnology, and SIRT2 (D4050, 12650S), SIRT6 (D8D12, 12486S), SIRT7 (D3K5A, 5360S), SIRT1 (D739, 2493S), SIRT3 (D22A3, 5490S) and Arf6 (D12G6, 5740) antibodies from Cell Signaling Technology.
Validation	FLAG-HRP (#A8592, RRID: AB_439702) and HA-HRP (sc-7392) were validated by blotting for FLAG and HA tagged overexpressed proteins.<br>ARF6 (3A-1, sc-7971), NMT1 (sc-393702), NMT2 (sc-136005), and SIRT2 (D4050, 12650S) showed decreased signal with knockdown and increased signal with overexpression of the target genes at the expected molecular weight.<br>SIRT6 (D8D12, 12486S), SIRT7 (D3K5A, 5360S), SIRT1 (D739, 2493S), SIRT3 (D22A3, 5490S) and Arf6 (D12G6, 5740) antibodies showed Western Blot bands of expected molecular weight and the signal decreased with the target gene knockdown.<br>Na/K-ATPase (sc-21712), TfR (CD-71) (sc-32272), and β-Actin-HRP (sc-47778) were validated by the manufacturer: increased signal was observed with overexpression of the target protein and immunostaining showed expected localization.<br>https://www.scbt.com/scbt/product/na-k-atpase-alpha1-antibody-c464-6?requestFrom=search<br>https://www.scbt.com/scbt/product/cd71-antibody-3b8-2a1?requestFrom=search<br>https://www.scbt.com/scbt/product/beta-actin-antibody-c4?requestFrom=search<br>TfR (CD-71) (sc-32272) was also validated in lab by colocalization immunofluorscence studies with transiently expressed TfR-mCherry and showed correct specificity.

Eukaryotic cell lines

Policy information about cell lines

Cell line source(s)	HEK293T (ATCC, CRL-3216)
Authentication	Authenticated by the vendor

Mycoplasma contamination	The cell line was tested negative for mycoplasma contamination
Commonly misidentified lines (See ICLAC register)	None
